# Cascade of care for hypertension among apparently healthy and unhealthy individuals of 25–64 years in the Czech Republic

**DOI:** 10.1371/journal.pone.0301202

**Published:** 2024-04-25

**Authors:** Barbora Berka, Michala Lustigová, Jana Urbanová, Pavlína Krollová, Ondřej Hloch, Alexandra Romanová, Juraj Michalec, Arian Taniwall, Kristýna Žejglicová, Jana Malinovská, Michael Jenšovský, Veronika Vejtasová, Juan P. González-Rivas, Geraldo A. Maranhao Neto, Iuliia Pavlovska, Jan Brož

**Affiliations:** 1 Department of Internal Medicine University Hospital Motol, Second Faculty of Medicine, Charles University, Prague, Czech Republic; 2 Department of Social Geography and Regional Development, Faculty of Science, Charles University, Prague, Czech Republic; 3 National Institute of Public Health, Prague, Czech Republic; 4 Department of Internal Medicine, University Hospital Královské Vinohrady, Third Faculty of Medicine, Charles University, Prague, Czech Republic; 5 Department of Cardiology, University Hospital Motol, Second Faculty of Medicine, Charles University, Prague, Czech Republic; 6 International Clinical Research Centre (ICRC), St Anne’s University Hospital Brno (FNUSA), Brno, Czech Republic; 7 Departments of Global Health and Population and Epidemiology, Harvard TH Chan School of Public Health, Harvard University, Boston, Massachusetts, United States of America; 8 Foundation for Clinic, Public Health, and Epidemiology Research of Venezuela (FISPEVEN INC), Caracas, Venezuela; 9 Department of Public Health, Faculty of Medicine, Masaryk University, Brno, Czech Republic; University of Oxford, UNITED KINGDOM

## Abstract

**Background:**

Despite accessible diagnostics and treatment of hypertension, a high proportion of patients worldwide remain unaware of their diagnosis, and even more remain untreated. Several studies suggest that absence of comorbidities is a predictive factor for unawareness and consequently non-treatment of hypertension. There are only a few studies that have assessed the hypertension prevalence and management among apparently healthy individuals.

**Objective:**

The aim of the study was to assess and compare hypertension prevalence, awareness, treatment and control among apparently healthy individuals, patients with internal diseases, and patients with non-internal diseases.

**Methods:**

Data from standardised blood pressure measurements conducted during the Czech European Health Examination Survey 2014 and information on health status and health care use collected during the Czech European Health Interview Survey 2014 were analyzed. We focused on participants aged 25–64. Data were weighted on sex and age to ensure an appropriate sex and age structure of the population. The χ^2^-test and binary logistic regression analyses were used to compare distribution of cascade of care for hypertension between the health-status groups of respondents.

**Results:**

The final eligible sample consisted of 1121 participants. The prevalence of hypertension was 32.8% in the healthy group, 29.8% in the non-internal disease group and 52.4% in the internal disease group, (p < 0.001). Awareness was 54.1%, 59.1% and 85.2% respectively, (p < 0.001). Treatment was 44.2%, 52.6% and 86.4%, respectively, (p < 0.001). Among apparently healthy respondents 62.6% had their blood pressure measured by a medical professional in the last year, compared to 71.1% in the non-internal disease group and 91.7% in the internal disease group, (p < 0.001). Differences in hypertension control were not statistically significant.

**Conclusions:**

Generally, our data show a discordance in hypertension management within the Czech population. Apparently healthy individuals are the least aware of their diagnosis and the highest proportion of them remain untreated.

## 1. Introduction

Hypertension is a serious medical condition with increasing prevalence worldwide. It is estimated that 31.1% of adults worldwide had hypertension in 2010 [1]. A pooled analysis of 1201 population-representative studies with 104 million participants from 2021 estimated an age-standardized hypertension prevalence of 32% in women and 34% in men in adults aged 30–79 [2]. As an important risk factor for cardiovascular diseases, which is the leading cause of death worldwide according to the World Health Organization (WHO), hypertension is a significant public health challenge [3, 4]. Each 20 mmHg rise in systolic blood pressure, as well as each 10 mmHg in diastolic blood pressure, doubles the risk of cardiovascular diseases [5]. In the Czech Republic, hypertension prevalence of 47% in men and 26% in women was found in the Czech European Health Examination Survey (Czech EHES) 2014, a cross sectional study in the population of people aged 25–64 years [6]. Since the detection of elevated blood pressure is both technically and economically accessible, hypertension can be effectively screened at the primary health care level in the Czech Republic. Also a number of effective medications is available to lower blood pressure and significantly reduce the risk of major cardiovascular disease events [7].

Awareness of hypertension is low, even in countries where healthcare is easily accessible. Additionally, a large proportion of patients aware of having hypertension remain untreated. Globally, 41% of women and 51% of men with hypertension remain undiagnosed [2]. In Europe the awareness of hypertension varies among its countries populations. The European Health Examination Survey (EHES) Pilot project conducted in 12 European countries in 2009–2012 found that 49% of men and 30% of women were unaware of having hypertension [8]. In the Czech Republic 40% of men and 24% of women are unaware of their condition according to Czech EHES 2014 [6].

Globally, only 38% of men and 47% of women with hypertension are treated and less than half of those treated had achieved hypertension control [2]. In the Czech population 48% of men and 62% of women with hypertension are treated according to the Czech EHES 2014 [6]. Even though the unawareness and non-treatment among hypertensive patients are high in the Czech Republic, the trend is positive according to Czech MONICA and Czech post-MONICA studies that assessed the prevalence, awareness and treatment between years 1985–2016/2017 [9].

To reduce the risk of cardiovascular disease events as a consequence of high blood pressure, the health care system needs to identify and target the unaware and untreated hypertensive individuals. Number of studies suggest an absence of comorbidities as a predictive factor for unawareness and non-treatment [10–12]. Healthier population groups seem to be at risk of hypertension unawareness and therefore in risk of delayed treatment. Few studies assessing the prevalence, awareness and treatment of hypertension among apparently healthy individuals can be found [13, 14].

This study is focused on hypertension prevalence, awareness, treatment and control with regard to patients comorbidities. The goal was to compare the cascade of care for hypertension within three population groups of ages 25–64 years in the Czech Republic—healthy individuals, patients with one or more internal diseases and patients with one or more non-internal diseases.

## 2. Methods

### 2.1. Study subjects

Data on hypertension obtained from the Czech EHES 2014 collected by National Institute of Public Health and information on health status and health care use obtained among the same individuals during the Czech European Health Interview Survey (Czech EHIS) 2014 collected by Institute of Health Information and Statistics of the Czech Republic were used [6, 15]. The EHES aimed to obtain current and relevant information about non-communicable diseases and their risk factors among the Czech middle-aged population [6, 16]. The study subjects were recruited among the study population of the Czech European Health Interview Survey (Czech EHIS), a cross-sectional survey conducted in all regions of the Czech Republic in 2014 with a representative sample of 6737 participants. It consisted of four modules on health status, health care use, health determinants and socio-economic background variables. The data was collected by professional interviewer-administered questionnaires. The EHIS study design including the complete set of variables obtained is available elsewhere [15]. Respondents (25–64 years old) of EHIS were further asked to participate in the EHES survey. Participation in the EHES survey was offered to 3850 respondents and 1220 participants were examined (response rate 32%). The EHES involved repeated measurement of blood pressure, anthropometric measures and blood sample analysis.

### 2.2. Ethical considerations

Written informed consent was obtained from all Czech EHES study participants. The Czech EHES study was conducted according to the applicable International Conference on Harmonisation (ICH) Good Clinical Practice (GCP) guidelines and the World Medical Association Declaration of Helsinki–Ethical Principles for Medical Research Involving Human Participants and was approved by The National Institute of Public Health of the Czech Republic.

### 2.3. Health outcomes

The main outcomes were prevalence, awareness, treatment, and control of hypertension within three study population subgroups—healthy participants, internal disease patients and non-internal disease patients. Participants were divided into the groups based on diseases they had suffered within the last twelve months prior to the survey. The diagnosis of hypertension was excluded from the group division criteria. Diseases from the EHIS 2014 questionnaire were divided into internal and non-internal diseases. The internal disease group consisted of diseases managed by internists; namely asthma, chronic obstructive pulmonary disease (COPD), myocardial infarction, ischemic heart disease, elevated cholesterol levels, stroke, diabetes, cirrhosis, renal diseases and thyroid disorders. Non-internal disease group consisted of arthrosis, arthritis, vertebral disorders, allergy, migraine, urine incontinence, depression and anxiety.

According to the health status, the participants were categorised into the three subgroups: “healthy participants,”“internal disease group” and “non-internal disease group.”The “healthy group”consists of individuals who reported no disease, the “internal disease group” consists of respondents who reported at least one internal disease and the “non-internal disease group” consists of subjects who reported at least one non-internal disease and no internal disease.

Hypertension was defined as systolic pressure >140 mmHg and/or diastolic pressure >90 mmHg and/or undergoing antihypertensive therapy. Hypertension control was defined as blood pressure values lower than 140/90 mmHg in patients taking antihypertensive medication [17]. During the health examination, systolic and diastolic blood pressure was measured using standard procedures according to the EHES Manual which describes the European level standards for national health examination surveys [18]. Three measurements were taken 1 minute apart after a 5-minute resting period. The values of systolic and diastolic blood pressure were counted as a mean of the second and the third measurement.

The information about the last year screening of blood pressure was obtained from EHIS questionnaire [15].

### 2.4. Statistical analysis

Data were weighted on sex and age to ensure an appropriate sex and age structure of the population. Descriptive statistics was used to characterise three health status groups in terms of sex, age, education and BMI category. The analysis of variance (ANOVA) was used to compare whether the means are significantly different or not (using the F distribution) in case of age and BMI. Further, χ^2^-test was used to compare the distribution of basic sociodemographic characteristics and distribution of BMI categories among the three defined health-status groups. Weighted prevalence of hypertension, awareness and treatment of hypertension across each health-status group and selected demographic variables are estimated and tested for differences. Firstly, the χ^2^-test was used to compare distribution of cascade of care for hypertension between the health-status groups of respondents. The differences in prevalence of hypertension (and its characteristics) within 1) healthy and internal disease group, 2) healthy and non-internal disease group, 3) non-internal and internal disease groups and finally 4) all three groups were tested. Secondly, because of the significant differences in structure among health-status groups, the binary logistic regression analysis models weighted and controlled for age, sex, education and BMI were used the estimate the chances of the hypertension prevalence, awareness, treatment, control. The chances in prevalence of hypertension (and its characteristics) are presented as odd rations (ORs) comparing 1) healthy vs non-internal disease group 2) healthy vs internal disease group, 3) non-internal vs. internal disease groups. STATA version 18.0 was used.

## 3. Results

A total of 1220 individuals were examined and interviewed in the study. From all the participants enrolled, incomplete data was obtained in 99 cases. The final eligible sample consisted of 1121 participants (91.9%), the mean age was 44.9 +/- 11.5.

Among the 1121 participants, 476 participants were healthy (42.5%), 322 patients were diagnosed with other than internal diseases (28.7%) and 323 patients were diagnosed with one or more internal diseases (38.8%). The mean ages of the healthy, non-internal disease and internal disease groups were 41.0 +/- 10.7, 42.0 +/- 10.7 and 50.5 +/-11.0, respectively and were statistically different (p<0.001). Body mass index (BMI) values of the healthy, non-internal and internal disease groups were 26.9 kg/m^2^ +/- 5.0, 26.8 kg/m^2^ +/- 5.3 and 29.4 kg/m^2^ +/- 6.3, respectively; p<0.001). General characteristics of the sample and the three subgroups with focus on sex, age, education and BMI are presented in Table 1.

**Table 1:** General characteristics of the sample and structure of healthy, non-internal and internal disease groups by sex, age, education and BMI category (sample weighted on age and sex).

*Characteristics/group*	*Total*	*Healthy group (1)*	*Non-internal disease group (2)*	*Internal disease group (3)*	*p-value* *
Total (N)	**1121**	**476**	**322**	**323**	
Mean age in years (±SD)	44.9 (±11,5)	41.0 (±10,7)	42.0 (±10,7)	50.5 (±11.0)	<0.001
Mean BMI in kg/m2 (±SD)	27.6 (±5,6)	26.9 (±5,0)	26.8 (±5,3)	29.4 (±6.3)	<0.001
**Basic characteristics of the groups (%)**					
**Sex**					0.002
Male	50.5	56.4	47.4	45.0	
Female	49.5	43.6	52.6	55.0	
**Age groups**					<0.001
25–44 years	51.2	64.2	50.9	32.3	
45–64 years	48.8	35.8	49.1	67.7	
**Education**					<0.001
Primary & Vocational	41.2	34.9	43.3	48.4	
Secondary	37.7	39.6	36.3	36.2	
University	21.1	25.5	20.5	15.4	
**BMI category**					<0.001
Normal	35.2	37.2	41.6	25.9	
Overweight	37.3	40.2	35.3	35.2	
Obese	27.5	22.6	23.2	38.9	

Note: *ANOVA was used to compare continuous data and χ^2^-test was used to compare categorical data.

The results of prevalence, awareness, treatment and control within the three groups weighted on age and sex are presented in Table 2.

**Table 2:** Cascade of care for hypertension in the three respondent groups–prevalence of health outcomes (sample weighted on age and sex).

Health outcome in % (N) / *Group*	*Total*	*Healthy group (1)*	*Non-internal disease group (2)*	*Internal disease group (3)*	*p-value* *			
Total (N)	1121	476	322	323	3 groups	1 vs 2	1 vs 3	2 vs 3
Hypertension (measured or treated)	37.5 (421)	32.8 (156)	29.8 (96)	52.4 (169)	<0.001	0.378	<0.001	<0.001
Awareness of HBP	67.7 (285)	54.1 (84)	59.1 (57)	85.2 (144)	<0.001	0.389	<0.001	<0.001
Treated hypertension	63.6 (267)	44.2 (69)	52.6 (51)	86.4 (147)	<0.001	0.196	<0.001	<0.001
Treatment among HBP aware	82.3 (234)	72.1 (61)	80.3 (45)	89.0 (128)	0.003	0.243	0.001	0.102
Control among treated (HBP < 140/90 mmHg)	43.2 (115)	45.2 (31)	46.4 (24)	41.2 (60)	0.756	0.965	0.552	0.559
Screening of BP by medical staff last year	73.4 (823)	62.6 (298)	71.1 (229)	91.7 (296)	<0.001	0.013	<0.001	<0.001

Note: * χ^2^-test was used to compare categorical data.

The prevalence of hypertension among the 1121 participants was 37.5%; 32.8% in the healthy group, 29.8% in the non-internal disease group, and 52.4% in the internal disease group, (p < 0.001). Among all the patients with hypertension, 67.7% were aware of the diagnosis. The awareness was lowest in the healthy group, where 54.1% were aware, compared to 59.1% in the non-internal disease group and a much higher awareness of 85.2% in the internal disease group, (p < 0.001).

Similarly, the proportion of hypertensive participants who were undergoing antihypertensive therapy was the highest within the internal disease group. Hypertension was treated in 44.2% of respondents in the healthy group, 52.6% of patients in the non-internal disease group and 86.4% of patients in the internal disease group, (p < 0.001). In total 63.6% of hypertensive study subjects were taking antihypertensive medication.

Among those aware of hypertension, 82.3% were treated. In the healthy group 72.1% patients were treated, compared to 80.3% in the non-internal disease group and 89.0% in the internal disease group, (p = 0.003).

Among the respondents with antihypertensive medication, 43.2% had their hypertension controlled). Sufficient hypertension control was found in 45.2% patients in the healthy group, 46.4% patients in the non-internal disease group and 41.2% patients in the internal disease group, (p = 0.756).

Based on the answers in EHIS questionnaires, 73.4% of participants did undergo a medical screening for hypertension in the year prior to the survey. Again, there are significant differences among the three groups. In the healthy group 62.6% of patients were screened for hypertension, 71.1% in the non-internal disease group and 91.7% in the internal disease group, (p < 0.001).

We are aware that the three groups differ in demographic characteristics and risk factors. Therefore Table 3 with logistic regression models weighted and controlled for age, sex, education and BMI is presented to eliminate the bias created through the demographic differences among the three groups. The results of logistic regression model support the findings received from χ^2^-test (Table 2), apart from two results comparing healthy and internal disease groups. The higher chance to have hypertension for respondents in the internal disease group compared to the healthy group is not statistically significant in the logistic regression model (p-value = 0.124). Also the chance of treatment among aware did not come out statistically significantly different between these two respondents groups (p-value = 0.055).

**Table 3:** Cascade of care for hypertension–chances of health outcomes (results of binary logistic regression models weighted and controlled for age, sex, education and BMI category).

	Healthy group (Ref = 1)			Healthy group (Ref = 1)			Non-internal disease g. (Ref = 1)		
	Non-internal disease group			Internal disease group			Internal disease group		
Health outcome	OR	95% CI	p-value	OR	95% CI	p-value	OR	95% CI	p-value
Chance of hypertension	0.72	0.49–1.06	0.095	**1.34**	**0.92–1.94**	**0.124**	1.85	1.25–2.74	0.002
Chance of awareness	0.91	0.49–1.68	0.766	3.50	1.92–6.34	<0.001	3.85	2.01–7.30	<0.001
Chance of treatment	0.99	0.54–1.81	0.988	3.22	1.85–5.62	<0.001	3.23	1.78–5.85	<0.001
Chance of treatment among aware	1.26	0.51–3.16	0.608	**2.24**	**0.98–5.11**	**0.055**	1.76	0.72–4.30	0.210
Chance of control among treated	1.10	0.49–2.49	0.813	0.80	0.41–1.58	0.528	0.72	0.36–1.45	0.370
Chance to be screened for hypertension last year	1.45	1.02–2.04	0.037	5.28	3.33–8.39	<0.001	3.65	2.25–5.93	<0.001

## 4. Discussion

The EHES study systematically evaluated the hypertension prevalence, awareness and treatment in the adult population (25–64 years) in the Czech Republic. Thanks to extensive data regarding the health status of all the participants, it was possible to compare the variables across the three defined population groups.

Our results show similar prevalence of hypertension among apparently healthy participants and patients with non-internal disease, compared to much higher prevalence in the internal disease group. Significantly lower awareness and treatment were found in healthy and non-internal disease groups compared to internal disease group.

There are only a few other studies assessing hypertension prevalence in healthy adult individuals. A study conducted among healthy individuals in Jeddah, Saudi Arabia, in May 2008 revealed a 22.8% prevalence of hypertension, which is significantly less than what our results showed in the Czech population. However, in Jeddah the number of study participants was rather small (n = 243) and they were selected randomly at a shopping mall without any further criteria ensuring a representative healthy population sample [13]. Another study focused on hypertension prevalence in healthy adults, a systematic review with data from 40 articles on the prevalence of metabolic syndrome among the apparently healthy workforce worldwide, published in 2019, revealed a hypertension prevalence of 33.7% consistent with our results. However, the cut off value for hypertension as a component of metabolic syndrome differs from the standard criteria for the diagnosis of hypertension (Systolic BP≥130 mm Hg or diastolic BP ≥85 mm Hg or treated for hypertension) [14].

As already mentioned in the introduction, number of studies have suggested absence of comorbidities as a predictor of poor awareness, which is in accordance with our results [10–12]. For example the French CONSTANCES population based study from 2020 found hypertension awareness to be predicted by female gender, age, prior cardiovascular disease, presence of diabetes mellitus, chronic kidney disease, level of education and higher BMI [11]. Similarly, a study from the Czech Republic shows higher awareness rates among diabetic patients with hypertension compared to the non-diabetic control group [19].

Regarding much lower awareness in the healthy and non-internal disease groups, one of the explanations for such difference might lie in hypertension screening. According to our data, the screening for elevated blood pressure targets the patients with internal diseases much more than others. While only 63.5% of healthy respondents and 72.7% of respondents with other than internal diseases had their blood pressure measured in the last year prior to the survey, 91.8% of respondents with internal diseases did undergo the screening. Respondents with internal diseases do have regular check-ups at specialists in various fields of internal medicine and are therefore more likely to undergo blood pressure measurement than patients who are either completely healthy or suffer from non-internal disease. Apparently healthy individuals do generally have a lower number of healthcare visits than patients treated for either internal or non-internal disease. A study from 2016 shows a higher number of healthcare visits as a predictive factor for better hypertension awareness [20].

However, in the Czech Republic, every health-insured citizen should undergo a regular screening checkup at their general practitioner every 24 months. Health insurance is compulsory for every permanent resident of the Czech Republic and a regular biennial screening checkup at general practitioner is then free of charge. According to Regulation on preventive care 70/2012 Sb., blood pressure measurement is a compulsory part of this checkup regardless of whether comorbidities are present [21]. Moreover, according to the Czech society for hypertension Recommendations for diagnosis and treatment of hypertension from 2022, higher awareness could be achieved if blood pressure was measured during every medical appointment, regardless of the doctor’s field of specialization [17]. Our results imply a need for strengthening hypertension screening in primary care in the Czech Republic. A systematic review assessing the level of detection of hypertension in primary medical care, conducted in 2018 revealed that levels of detection are around 60% [22]. Interesting data on hypertension management in primary care has been presented in an Italian study from 2014 on a sample of 6717 hypertensive patients. According to the study results, in 1769 of them no blood pressure measurement was performed by a GP in the last year [23]. A low awareness of hypertension in the Czech Republic was also described in a cross-sectional study comparing among others hypertension awareness in the Czech Republic, Poland, Russia and Lithuania. It shows that the proportions of having self-reported high blood pressure were the lowest in the Czech Republic. [24]. However, the trend in hypertension awareness among Czech men and women is positive. According to Czech MONICA and Czech post-MONICA, studies that monitor the trends in major cardiovascular risk factors over the past 30 years in the Czech population, awareness of hypertension increased in both sexes [9].

We found very low treatment in the healthy group and the non-internal disease group as well. This is partly caused by lower awareness in these groups, but not solely. Treatment among aware is also lower than in the internal disease group. This implies that either the primary care providers do not give enough importance to hypertension treatment in patients without internal disease comorbidities or that the compliance of these patients is low.

The results suggest no significant difference in hypertension control within the three groups. Since the analysis did not prove the differences in control to be statistically significant, further research should be conducted on hypertension control within apparently healthy individuals.

## 5. Strengths and limitations

Since the data and sample collection were done under the guidelines and the requirements for the implementation of standardised national health examination surveys (HES) in European countries, the study results can be compared to the findings of similar studies in the region. Also the study brings original data on hypertension prevalence, awareness and treatment within population groups that had not been studied much before.

However, the response rate of the EHES survey was 32%. The most frequently cited reasons for non-participation beyond lack of interest were: lack of time, problematic availability of the examination site, completion of preventive check-ups with their GP or regular specialist visits. Furthermore, non-respondents reported being ’healthy’ or having fear of being diagnosed. In particular, these arguments indicate a low level of health literacy of some non-respondents [8]. Several studies have shown a lower level of self-rated health or higher prevalence of chronic diseases among non-respondents, therefore our results on hypertension prevalence could be an underestimate due to selection bias [16, 25, 26]. In recent decades, population health surveys have tended toward lower response rates not only among the Czech population, but also in other countries [9, 16, 27, 28].

## 6. Conclusion

Our results show low hypertension awareness and treatment among apparently healthy individuals. Regular attendance at a medical office of other than internal medicine specialists didn’t show a significant improvement in hypertension detection or treatment. However, the awareness and treatment were proven to be significantly better among patients with other internal diseases.

This implies the need for more effective primary medical care hypertension screening in order to target apparently healthy individuals and patients of doctors from fields outside internal medicine. As stated in the Czech hypertension society’s Recommendations for diagnosis and treatment of hypertension from 2022, higher awareness could be achieved if blood pressure was measured during every medical appointment, regardless of the doctor’s field of specialisation.
